# *‘We Keep It Secret So No One Should Know’* – A Qualitative Study to Explore Young Schoolgirls Attitudes and Experiences with Menstruation in Rural Western Kenya

**DOI:** 10.1371/journal.pone.0079132

**Published:** 2013-11-14

**Authors:** Linda Mason, Elizabeth Nyothach, Kelly Alexander, Frank O. Odhiambo, Alie Eleveld, John Vulule, Richard Rheingans, Kayla F. Laserson, Aisha Mohammed, Penelope A. Phillips-Howard

**Affiliations:** 1 Liverpool School of Tropical Medicine, Liverpool, Merseyside, United Kingdom; 2 Kenya Medical Research Institute/Center for Disease Control Research and Public Health Collaboration, Kisumu, Nyanza Province, Kenya; 3 Safe Water and AIDS Project, Kisumu, Nyanza Province, Kenya; 4 Centre for African Studies, University of Florida, Gainesville, Florida, United States of America; 5 Center for Global Health, Centers for Disease Control and Prevention, Atlanta, Georgia, United States of America; 6 Division of Reproductive Health, Ministry of Public Health and Sanitation, Nairobi, Kenya; University of Oxford, Kenya

## Abstract

**Background:**

Keeping girls in school offers them protection against early marriage, teen pregnancy, and sexual harms, and enhances social and economic equity. Studies report menstruation exacerbates school-drop out and poor attendance, although evidence is sparse. This study qualitatively examines the menstrual experiences of young adolescent schoolgirls.

**Methods and Findings:**

The study was conducted in Siaya County in rural western Kenya. A sample of 120 girls aged 14–16 years took part in 11 focus group discussions, which were analysed thematically. The data gathered were supplemented by information from six FGDs with parents and community members. Emergent themes were: lack of preparation for menarche; maturation and sexual vulnerability; menstruation as an illness; secrecy, fear and shame of leaking; coping with inadequate alternatives; paying for pads with sex; and problems with menstrual hygiene. Girls were unprepared and demonstrated poor reproductive knowledge, but devised practical methods to cope with menstrual difficulties, often alone. Parental and school support of menstrual needs is limited, and information sparse or inaccurate. Girls’ physical changes prompt boys and adults to target and brand girls as ripe for sexual activity including coercion and marriage. Girls admitted ‘others’ rather than themselves were absent from school during menstruation, due to physical symptoms or inadequate sanitary protection. They described difficulties engaging in class, due to fear of smelling and leakage, and subsequent teasing. Sanitary pads were valued but resource and time constraints result in prolonged use causing chafing. Improvised alternatives, including rags and grass, were prone to leak, caused soreness, and were perceived as harmful. Girls reported ‘other girls’ but not themselves participated in transactional sex to buy pads, and received pads from boyfriends.

**Conclusions:**

In the absence of parental and school support, girls cope, sometimes alone, with menarche in practical and sometimes hazardous ways. Emotional and physical support mechanisms need to be included within a package of measures to enable adolescent girls to reach their potential.

## Introduction

Adolescence is a critical period of psychological and biological change, and emphasis has shifted in recent years to enhance interventions that protect young peoples’ lives [1]. However, knowledge on the health and wellbeing of adolescents in low and middle income countries (LMIC), and the social determinants that impact on this, is limited [1], [2]. Evidence of associations between education and health has strengthened international resolve to focus attention on improving educational opportunities for adolescents, particularly girls [3]. Staying in school potentially protects girls against early marriage, teen pregnancy, and other reproductive and sexual harms including HIV infection [4]–[7]. The burden of these harms among adolescent girls is disproportionately high in sub-Saharan Africa (SSA) [8]–[10].

Menarche and menstrual management is thought to contribute towards inequity in schooling [11], [12]. While menstruation is a normal bodily function and attitudes to it vary substantially by culture [13], [14], it is often seen as contaminating [15], or severely debilitating [16]. Among adolescent girls in high income countries, it is often considered shameful and embarrassing, despite an adequacy of facilities and information [17]–[20]. There are fewer studies on girls’ attitudes to menarche and menstruation in SSA [21], with recent studies reporting girls are shamed by stained clothing [11], [22]–[24]. Such studies have suggested poor menstrual management thus causes girls to miss school [11], [12], [23], with calculations made that a girl will miss 10–20% of her school days [25]. In Tanzania, a study raised concern that menarche may also place girls at risk of sexual advances [12].

Interventions which provide better menstrual management to schoolgirls thus may have a role in improving schooling, equity, sexual and reproductive health, and wellbeing [11]. Small-scale studies to date, however, have not generated unequivocal evidence that improving menstrual solutions reduces school absenteeism or risks of sexual harm [24], [26], [27]. As part of a baseline evaluation for a randomised controlled pilot study examining the potential benefits of different menstrual solutions for adolescent primary schoolgirls in rural western Kenya, we have examined girls’ attitudes, experiences, and concerns around menarche and menstruation.

## Methods

### Study Area and Population

The area under study is a site within the health and demographic surveillance system (HDSS) of the KEMRI/CDC Research and Public Health Collaboration [28], [29], in Gem District, Siaya County. This rural district lies in the north-western part of Nyanza Province, 400 km west of Nairobi. The population are almost exclusively members of the Luo ethnic group, mostly Christians, who are occupied mainly as subsistence farmers and fisherfolk [29]–[31]. It is a polygynous society [30]; typically, each wife lives in a separate house with young children, while older children and adolescents live in separate houses.

While Nyanza is one of the poorest provinces in Kenya, it has a literacy rate of 70%, compared to the national average of 62%. National surveys estimate four out of ten child learners miss school daily in Siaya County [32]. The gender equity seen in schools at ages 12–13 years widens during later adolescence with 23% more boys than girls attending school by 18 years of age [33], [34]. The area typifies the disease burden of rural African communities [28]. Mortality among adolescents and young people is predominantly attributed to communicable diseases and injuries [35]. Nyanza Province also ranked highest amongst all provinces in Kenya on measures of physical and sexual violence against females, with 12% of women reporting their first sexual intercourse was against their will [36].

### The Menstrual Solutions Study

This research is nested within the Menstrual Solutions Study (Ms Study), a single-site three-armed open cluster randomized proof of concept study; with school the unit of randomization and schoolgirls the unit of assessment. Following meetings with head teachers, chiefs, and medical and educational officers, 30 eligible schools were randomized into three arms, with girls in the target age group of 14–16 years to receive sanitary pads, menstrual cups, or usual practice. Girls were required to have had a minimum of 3 menstrual periods to be eligible for the study. Participating girls have been followed longitudinally to examine acceptability, use, and safety of menstrual products, and to explore the contribution of menstruation to school absenteeism and engagement.

### Recruitment and Participants

Baseline focus group discussions (FGDs) were held at eligible Ms Study schools, and included girls in all three arms of the study. All head teachers were requested to allow their girls to participate in the baseline qualitative study. Of 30 schools randomised for the study, a random selection of 6 schools was taken, with an equal proportion (2 schools) from each arm. A member of the research team visited the schools, to provide information on participation in the focus group discussions to girls who were eligible to participate in the wider study. In all but one of the primary schools, the number of eligible girls were relatively few (girls had either not reached menarche, or had not yet experienced three periods). All eligible girls strongly voiced a wish to participate in a FGD and were therefore given the opportunity to do so. In one school there was a high number of eligible girls, all of whom were again, particularly keen to take part in a FGD. The decision was taken to have 2 separate FGDs in this school so that all girls could be included. Following written consent by the girls, their parents were then visited at home to request their approval, and provided written consent for their daughter to participate in the baseline FGD at their school. A review of transcripts from the initial FGD indicated that saturation had not been reached, with new themes emerging from the data which required further exploration. These themes were identified and added to the FGD guide. Four additional FGDs were then conducted until we reached saturation. A similar sampling methodology to the above was used.

### Focus Group Approach

All FGDs were conducted by a moderator and a note taker. Both were young females who were Luo and local to the study area. They were fluent in both Luo and English. One of the researchers acted as observer during several of the FGDs and at the conclusion of the FGDs discussed the results and issues raised with the moderator and note taker in order to develop a closer understanding of the data. All discussions were conducted in Luo and lasted approximately 1 hour 45 minutes including introductions, ground rules and procedures for consent. A focus group guide was developed to standardise FGD across groups, consisting of questions around girls’ experience and understanding of menarche, and menstruation. The semi-structured guide comprised the following topics: experience of menarche, experiencing menstruation, normal menstrual practice and hygiene, and laundry at home and school. All FGDs were tape- recorded with notes taken to capture the main points, group dynamics and non-verbal gestures. The discussions were transcribed verbatim and translated from Luo into English. The transcripts were then reviewed for accuracy by the moderator.

### Analysis

The data were analysed using thematic content analysis [37]. The English language transcripts were read several times by two senior researchers for familiarity with the raw data and to set up an initial skeleton coding frame using the key themes which emerged. The coding frame comprised the major issues including knowledge of (and preparation for) menstruation, experience of menstruation, menstrual practices, schooling, and other experiences associated with menstruation. Each transcript was then coded by the lead researcher according to the framework, with additional codes added where data indicated an emerging theme. The raw data were revisited time and again, to reflect on the interpretation of the narratives. The framework was divided further into subthemes. The narratives were then recoded where relevant, under these subthemes. Another senior researcher studied the completed coding frame and quotes to check for consistency, interpretation and to add any additional themes/sub themes where necessary. Only minor differences were noted, which were discussed and agreement reached after revisiting the data. Further analysis was undertaken by noting strength of feeling, use of adjectives, and frequency of quotes within each theme/subtheme, contradictions or agreements across and within the different groups. The data for each theme and subtheme were then weaved together to provide a textual commentary.

As part of the wider study, six FGDs were conducted with 57 parents and members of the community to explore cultural views around menstruation and any challenges of introducing the menstrual cup to young girls in this population. The participants were recruited at village meetings where information was provided about the FGDs by field staff and informed consent was taken from those wishing to participate. All of the parents who consented then participated in the FGDs which were held in school compounds at a later date. The participants were recruited at community meetings set up to introduce the study to the local population. A request was made at each meeting for volunteers to participate in a FGD to be held at a later date. These FGDs were carried out using the same moderator and note taker as for the girls FGDs and analysed by the same authors using a similar framework for consistency. Although conducted separately, with different questions asked than for the girls FGDs, some of the same themes emerged, showing similarities with data from the girls’ narratives. These have been incorporated in to the paper to provide data triangulation. Quotes below provide FGD and participant number, except where the note-taker was unable to capture who spoke (notated as ‘x’).

### Ethics

The Ms Study was granted ethical approval from the Scientific and Ethical Review Boards of the Kenya Medical Research Institute, the Institutional Review Board of the U.S. Centers for Disease Control and Prevention, and the Ethics Committee of the Liverpool School of Tropical Medicine. Parents provided written consent and girls gave their written assent.

## Results

In total, 120 girls took part in 11 FGDs. The groups ranged between 7 and 13 participants each. The majority of participants (80%) were 14 years of age, 16% were aged 15, and 4% were aged 16 years (Table 1).

**Table 1:** Focus Group Participants.

School	Age 14 y	Age 15 y	Age 16 y	Total
A	9	2	1	12
B	12	–	–	12
C	6	4	1	11
D	5	3	1	9
E	12	1	–	13
F	9	2	1	12
G	9	2	1	11
H	8	–	1	9
I	6	1	–	7
J	12	–	–	12
K	8	4	–	12

As girls were given the opportunity to raise issues considered relevant to their menstrual experiences the following themes emerged: lack of preparation for menarche, maturation and sexual vulnerability, menstruation as an illness, secrecy, fear and shame, coping with inadequate alternatives, paying for pads, and struggles with menstrual hygiene. The main findings from the discussions are presented below.

### Lack of Preparation for Menarche

It was very common for girls to report no prior knowledge of menstruation, describing learning of menstruation only when they experienced bleeding for the first time. Many parents also recognised that they had not prepared their daughters for menstruation.

‘*When I first started my lower abdomen was stretching painfully and when I went back to the classroom everybody was asking me what was on my clothes, then I told them I don’t know’*. (P2 School H).

*‘What I can say is that (cough) these girls are scared because they think that they are the only ones experiencing monthly period. And also it is our fault as parents not to share with our girls of the changes they will be undergoing. They are not aware that all females undergo the same.’ (P8 Parent FGD 4)*.

Consequently, menarche appeared to be a ‘*shock*’ to some, while other narratives were very matter of fact.

*‘I was going to the latrine and I also had stomach cramps then I was surprised to see blood coming out’; Moderator ‘What did you do?’; P2 ‘Nothing, I just went back and changed my pant.’* (P2 School J).

Girls were questioned about the context of menstruation in Luo culture. The girls responded that it was not celebrated across the community, nor within individual households but rather it was kept secret sometimes even from a girls’ own mother. Mothers mentioned old traditions of advice from older women no longer occurred.

*‘That’s why they get pregnant while still young since they had already started menstruating and know it among themselves and don’t even inform the parents.’* (P7 Parent FGD3).

The few girls who did receive information prior to reaching menarche reported that it was rarely more detailed than they will bleed every month from their vagina when they reach a certain age, and that ‘*timo timbe maricho’* (*‘*doing bad things’) once they had started ‘*attending*’ could lead to pregnancy.

*‘My mother told me that if I reach thirteen years I will start experiencing monthly period.’* (P8 School J).

‘*I saw blood from my vagina and I never knew anything. I went to my mother and I told her what I saw and she told me that is my monthly period. I should be careful.*’ (P5 School D).

‘*He [uncle] told me that people start menstruating at the age of eight. Then he also told me not to allow myself to be deceived by men because I can get pregnant at that time.’* (P5 School C).

Information about menstruation was usually given to girls by females; their mothers, older sisters or their fellow pupils. One girl described being told by her uncle, with whom she lived. Some girls had been taught in science class, and both female and male teachers were reported to have responsibility for this. Despite strong views on the need to maintain secrecy about menstruation from males in particular, there was no negative feedback from girls who were taught by a male.

When asked by the moderator what menstruation is, generally girls gave a rudimentary or confusing description, illustrating their limited understanding of their bodies.

*‘The blood comes from the ovary, then it comes and breaks into the uterus. Then it becomes blood and that is the monthly period.’* (P2 School J).

*‘You see blood.’* (P10 School G).

*‘Yes what blood is it?’* (Moderator School G).

*‘Fertilised blood from the vagina.’* (P7 School G).

### Menarche, Maturation and Sexual Vulnerability

On reaching menarche most girls saw this as a sign of being grown up or mature.

*‘I feel that I am now a grown up because once you have started your menses, when you like playing with men you can become pregnant.’* (P9 School H).

However, not all girls agreed. Some felt they were neither an adult nor a child, others felt they were still a girl, although noting that they were now less likely to play with other children. Girls also appeared to receive mixed messages about how they were viewed by others.

*‘I do feel that I am neither a child or an adult.’* (P4 School H).

‘*One day she [mother] found me in the kitchen and I had not lit the cooking fire and she told me that I was a grown up and I should have lit the fire… and my grandmother told her that am not a grown up.’* (P2 School J).

In line with the possibility of conceiving, menstruation was perceived by girls and those around them, as a sign they had reached sexual maturity. Girls volunteered that starting menses also meant that they became sexually attracted to boys.

‘*When we start menstruating we long for some things.’* (P12 School E).

‘*But what are these things that we do long for?*’ (Moderator School E).

*‘You long to have sex with a boy.’* (P12 School E).

Boys were also reported to become aware of girls sexual maturity at around this time, although this was because they noticed the girls’ growing breasts and hips. Their awareness meant they put girls under pressure to have sex.

‘*If boys want to know that you are old enough to have sexual intercourse, he looks at your breast. After noticing that your breast has enlarged, he will tell you “I long for something can you give me.’* (P7 School D).

Serious issues around starting menses and subsequent sexual maturity were raised in four of the FGDs, although this was only mentioned by one or two girls in each of the four groups. These participants suggested that girls were seen as either now mature enough to be married off, or they were at potential risk of sexual abuse, including from relatives, such as their fathers. This issue was also raised by one of the mothers in a parent FGD.

*‘I think when a girl starts her monthly periods I restrict her from moving around with the father because he might take advantage of her.’* (P8 Parent FGD3).

*‘They (men) see that, as in, they see your breasts are enlarging,(laughter) pimples appear on your face, and hair also grows on pubic area, hips broadening, and so you are mature to marry.’* **(**P4 School I).

‘*We fear telling father. He can have a negative thought. He can have another mind?’* (P6 School B).

*‘Which kind of mind?’* (Moderator School B).

*‘He can take advantage of you. He can think that my daughter is now a grown up, sowhen you are left alone with him in the house he can sleep with you.’* (P6 School B).

*‘There are some [fathers] if you tell them will say you are now mature and they can even rape you if you don’t have your mother around’.* (P6 School D).

### Menstruation as an Illness

The experience of menstruation itself was associated with a core group of physical symptoms commonly mentioned within all groups, as well as emotional symptoms. Consequently, *‘you feel like somebody who is ill.’* (P7 School D). Physical symptoms included headache, stomach ache, backache, and tiredness. Stomach ache was universally reported by girls, often described as ‘*stretching’* of the lower abdomen. Less commonly mentioned were dizziness, nausea and vomiting and restlessness. Housework became more difficult for some, although most girls still continued doing their usual chores. Only one group reported that if they told their mothers they were ‘*not well’* they were told to stay at home and rest. Some girls described emotional effects of menstruation including boredom, being moody, feeling lonely or shy and not wanting to talk to others.

‘*I always feel, you are lonely all the times, you feel the blood coming out rapidly in drops. During that time you become tired, you feel headache and you become restless and you cannot work, even if you do some chores, you are just dull.’* (P3 School G).

### Secrecy

Narratives repeatedly mentioned the importance of secrecy, a recurring theme throughout the FGDs that *‘it is something that we hide’*. This was also emphasised by parents as customary.

‘*In our community we have never talked about it because we feel ashamed.’ (P8 Parent FGD 3).*

‘*Blood is something so secret that it is not recommended anyone to see.’* (P4 School K).

*‘You can have fear because sometimes you live with your father, and at times you cannot share with your father these things.’* (P1 School B).

*‘You keep it secret? Why do you keep it secret?’* (Moderator School H).

*‘We keep it secret so that no one would know.’* (P6 School H).

*‘Who should not be aware?’* (Moderator School H).

*‘My classmates.’* (P6 School H).

Few girls across all groups felt they could ‘*share*’ with another female. Those who did usually shared with their mother, or sometimes with another close relative (aunty or grandmother), female teacher or friend because they experience the same issues and can therefore help. However, some girls felt it was even too private to share with their mother.

*‘Some mothers are not the same, there are some who even take alcohol if you ask her for money she will tell you that is something she doesn’t know and you are disturbing her and then she would rather take the money to buy alcohol, so you will just look for ways of helping yourself and how you can keep clean while going to school.’* (P1 School B).

Most girls were of the opinion that males – fathers, brothers or classmates should not be aware that they were menstruating. This is because they are ‘*not supposed to know*’, or from a general ignorance about the subject so that: ‘*Your father sometimes is not even aware that girls do have monthly period’.* It was important to most girls that boys remained unaware in order that they would not laugh at them and tell other people (although teasing was not limited to boys as girls reported that their female peers also joined in).

### Fear and Shame

Fear, the prevailing emotion associated with menstruation in each focus group, and a major reason for the need for secrecy, was not directly associated with knowing a girl was ‘*attending*’, but mainly because of the shame surrounding leaking blood, or keeping clean. The girls dreaded leaking, a shameful event, because it resulted in stained clothing, causing others to laugh and tease them of their predicament. All discussions echoed concerns over the fear of soiling, with narratives also describing actual occurrences rather than just a fear.

‘*Even if I come to school I am embarrassed because at times I don’t have what to use, or if I have cloths maybe I’ve not worn them properly or I’ve only worn my underwear so when I get up my dress is already soiled*.’ (P4 School H).

Fear of leaking at school, in particular, was a dominant theme; partly because of the practice where pupils stand up to respond to a teacher, thereby revealing their soiled dress. Coping strategies included tying a sweater around the waist, seeking permission from a teacher to bathe or to go home, hiding until a friend could help them, or going home without permission. If they were able to confide, friends helped by approaching the teacher for permission or by getting them water to clean themselves. Despite the support, girls still voiced a general fear of ‘friends’ seeing their blood-stained clothing, and the resulting shame. Although it was not clear if the girls who went home to bathe or change returned to school that day, it was acknowledged by both girls and parents that girls missed school during their menstrual cycle, either because of lack of items to manage their menses, or due to physical symptoms.

*‘What about other girls, do they take time off or just come to school as usual?’ (Moderator School F).*

*‘They can absent themselves from school.’* (P11 School F).

*‘They can absent themselves from school because of the materials that they are using and so they get embarrassed.’* (P3 School F).

*‘Sometimes they have painful stomach cramps that walking becomes a problem and also lack of things to use to manage menses because the parents cannot afford pads.’* (P5 School C).

*‘Most of the girls do not go to school during their monthly periods, because as Luo’s we still cling to the past. We cannot buy our children proper materials for managing their menses even a cotton wool. We tell them to wear old pieces of clothes instead. They then become fearful and feel that when they get up the clothes might come out and so her classmates or the teachers might laugh at her, so much embarrassments make them not to go to school during their periods.’* (P1 Parent FGD 1).

Most of our participants, however, insisted that they themselves went to school at such times but their classmates might not. Only girls in one FGD admitted they missed school.

‘*My mum can’t afford to buy me what to use during menstruation and if I use cloth I might get soiled so I don’t want to be embarrassed.’* (P9 School C).

However, when girls attended class during their menses, they had to cope with many issues, including that their teachers may not be understanding or helpful.

*‘You can fear asking for permission from the Teacher to go change in the latrines.’* (P7 School H).

They also found it difficult to concentrate in class because of the fear of leakage, and/or fear of odour from the leaking menstrual blood.

‘*Sometimes when I am in class and the teacher is teaching, I don’t concentrate on what is being taught because your mind is always on the thought that when you stand and your clothes will be blood stained and the teacher will see, hence you don’t concentrate.’* (P3 School H).

‘*You feel you do not want to stay in class, all you feel is that you should go back home and rest because you cannot concentrate in class even if the teacher is teaching. You might be thinking that your dress might get soiled.’* (P10 School G).

‘*You do not want to sit next to your teacher, maybe you are smelling.’* (P2 School G).

Punishment by keeping girls at school either during lunch, or after school, had distressing consequences for menstruating girls:

‘*Sometimes you were punished in school and you were unable to go home for lunch and maybe you were using cloth so you had to wait until evening. In the evening you will find your cloth soiled and your thighs bruised due to friction made by cloth, so you can’t manage to walk, your thighs are very painful, you can’t walk unless you take off that thing.’* (Px School D).

*‘It is always difficult because sometimes you are going to school and you know very well that during lunch hour you will be going home to bathe and sometimes you are punished so you are unable to go home and bathe so you will be forced to stay that way till evening.’* (P6 School D).

Caning was also mentioned with girls reporting their wish to be able to offer the hand rather than the buttocks for being beaten.

### Coping with Inadequate Alternatives

Commonly, girls described *themselves* as using pads but their peers as using different products, although one girl summed this up thus: ‘*Some people are saying they are using pads but they are using cloths, majority uses cloths, some mattresses’.* (P1 School B). Pads were clearly a preferred and valued item, and although obtainable, the price meant they were either unaffordable or rationed for a significant proportion of the girls. The most commonly mentioned alternatives were old clothes, blanket or pieces of (bedding) mattress. Some girls used several pairs of panties, socks, towel, cotton wool or tissue. Very occasionally mention was made of using grass, leaves, polythene, paper or material from sacks. When girls’ menses start unexpectedly, grass or leaves plucked from the ground around the schoolyard was reported to be the only solution available for them.

‘*When this thing [periods] comes suddenly while you are in school and you do not want people to notice you, and you did not carry even pieces of blankets, you can look for ways to manage it in vain, so you can take grass that is not very dry, mix with leaves then use to prevent leakage.’* (P3 School F).

As well as being prone to leakage, such items were also perceived to be associated with problems of infection and causing other harms:

*‘Some cloths might infect you. For instance if you use damp cloths you might start itching then it turns to a wound.’* (P1 School C).

‘*It can hurt you, if someone step on grass first – you will again use the same grass to manage your menses, it will make you sick*. *You can even contract malaria, isn’t it?’* (P3 School F).

‘*When you are using mattress it can cut into small pieces and it can get inside the vagina.so she can get problem in the vagina*; (P9 School D).

‘*She can get cancer’*. (P3 School D).

*‘You feel pain when you are urinating.*’ (P7 School D).

*‘Vaginal ulcer*.’ (P1 School D).

Other consequences of using these alternatives included a fear of it dropping the menstrual item, mentioned in six of the discussion groups. This constrained the girls due to physical discomfort, affecting their ability to engage in their normal school activities, reported as running, playing and even just walking. These issues were also described during parent FGDs.

‘*There are challenges because when you wear cloths, you will have to walk; you will feel that the cloth might come out before reaching your destination, so you become fearful.’* (P3 School I).

*‘Sometimes she doesn’t go to school due to the bruises she got from using blanket.’* (P10 Parent FGD 3).

*‘Pain’, ‘harm’, ‘hurt’, ‘irritation’, ‘bruising’* were words used frequently to describe the consequences of wearing such items. This was either as a direct consequence of the product itself that was being used, or because of the length of time such items had to be worn before they could be replaced.

*‘Using pieces torn from mattress… when you use them your thighs get irritated, you can’t even walk comfortably.’* (P4 School D).

Many girls across all groups spoke of using pads, (one brand in particular) which were comfortable, not associated with any specific challenges, and ‘*not easy to stain your clothes*’. Some girls mentioned that one pad was supposed to be worn for an extended time (8 hours).

‘*Let me say if you wear pads in the morning at 7, you are supposed to take 8 hours, not that you are wearing it in the morning, then you change in the evening. You will find it has dried up, that it won’t be easy to remove it, you will feel pain.’* (P6 School B).

Rationing of pads to make them last longer resulted in discomfort. A few girls acknowledged that they didn’t always have pads, or enough pads, so would on these occasions have to wear cloth. Just one girl stated she preferred cloth which she could wash after using rather than throw away a manufactured pad.

### Paying for Pads

Branded sanitary pads, or money to buy these pads, was mostly given by mothers; although other relatives were mentioned, including fathers, uncles and brothers. Some were often not aware of what the girls were purchasing with their money. Three of the groups described how boyfriends sometimes gave money to their girlfriends, or that boyfriends were asked for money which was then used to buy items such as pads. This was also reiterated by some parents.

‘*You cannot be bold enough and tell him that you want to buy pads. You will tell him that you need the money to buy something?’* (Moderator School F).

*‘If you tell him that you are going to buy pads, then he will not give you money?’* (P12 School F).

*‘He will ask you what pads are.’* (P12 School F).

‘*Where do girls get pads?’* (Moderator).

*‘From parents or else it will force her to have a boyfriend to buy her pads because she fears using cloth due to leakage. I would just urge parents to buy their daughter pads so that they don’t engage in early sexual activities.’* (P2 Parent FGD 4).

As mentioned by the parents as above, asking money for pads comes at a cost.

‘*What does she give the boy in return?’* (Moderator School F).

*‘Her body.’* (Px School F).

‘*Her body, how is that possible? How will she give her body?*’ (Moderator School F).

‘*You pay him your vagina.*’ (P5 School F).

‘*You play sex with him.*’ (P4 School F).

‘*So does it mean that when given money, they expect sex in return?*’ (Moderator School F).

‘*Yes.*’ (P-all School F).

This narrative was not unique to this one school, with similar responses by girls in other schools.

‘*Some people exchange sex for money. The money is used to buy pads. Maybe she is being given money then they have sexual intercourse… sometimes is good, sometimes it’s not because you need help, so you will just engage yourself into sex.’* (Px School C).

There were inferences in one FGD that acquisition of money for the household from having sex was tolerated and perhaps condoned by some parents.

*‘Some parents play a great role in making their girls engage in early sex because their girls sometimes spend from morning to evening without coming back home, or they don’t come home at all, then when she comes with money and gives to the parents, they don’t question where they got it from.’* (P6 School C).

Comments from the parent FGDs confirmed both that they were aware that boyfriends gave girls money which they used to buy pads with, and also they knew that some girls had sex in exchange for money.

*‘She will go look for this money from the men, and that’s how they can end up with the unwanted pregnancies.*’ (P2 Parent FGD 1).

### Struggles with Menstrual Hygiene

Issues around hygiene were also explored with narratives demonstrating challenges with bathing, and washing. Sometimes girls had to compete with family members for scarce resources such as soap and water, which could cause conflict, even with mothers.

‘*Sometimes you want to take a bath three times and your mother quarrel’s you that you are misusing her soap.’* (P5 School J).

*‘Your parent will wonder why her daughter is bathing three times which she never does daily, and maybe you didn’t fetch water for bathing so you will be forced to use water that was stored in the house and she might say you are misusing her water.*’ (P6 School D).

In addition to using scarce household resources, frequent bathing made it harder for girls to conceal when they are menstruating. If ‘*seen taking bath severally*’ (P9 School J), it is a cause of shame because people then ask why they bathe frequently. Lack of soap and water resources also impacted on keeping underwear or menstrual items clean.

*‘Maybe you only have one underwear that you wash and wear.’* (P2 School G).

*‘You can develop a stench…when there is no soap, you become stressed up because you don't know how you will wash them.’* (P7 School H).

The act of washing bloody, soiled cloths was also a cause of stress and shame for girls, and vocalised as an issue for girls’ perceived self-esteem.

*‘I feel bad because I might be washing it where people are and also flies might be following me where am washing them and I will be ashamed.*’ (P8 School J).

Washing of items could be straightforward, if facilities such as a bathroom were available. None of the girls in the FGDs mentioned water as scarce at school, although soap was rarely available. Some girls did not wash items at school but instead ‘*They replace the used one with a fresh one and then they look for something to keep the used one which they will wash back at home.*’ (P9 School J). That led to concern that the item might fall out of their pocket and be seen by others. Drying items could be equally difficult as this was supposed to be carried out in private. This took place anywhere private, including the bedroom, bathroom, on an outside airline (although not one commonly used), or in front of the house. Clandestine approaches were used to maintain secrecy with a few girls describing how they dried cloths on the washing line late at night, and then got up early to retrieve the articles before others woke up. Another girl mentioned drying items underneath her clothes so that they remained hidden from view.

Disposal of pads was primarily inside the pit latrine, and seemed straightforward for the majority of girls, although not all.

‘*After using [brand name] you do not dump it in the latrine, or burn them, you throw some place where nobody can see.’* (P4 School H).

Cloth, blanket, and mattress were also sometimes dumped in the latrine, or occasionally burnt, buried, or just ‘*thrown*’. A couple of girls mentioned this menstrual practice to be irresponsible.

‘*You’re not supposed to throw it if you have two, because you can wash and reuse it. And if you throw them anywhere your younger ones might find it and they may play with it and that’s not good.’* (P1 School C).

## Discussion

To date, few qualitative studies have explored what it is like for young adolescent girls to manage their menses in sub-Saharan Africa. McMahon et al [23] questioned girls aged 12–16 years on their methods for managing menstruation, and the difficulties they faced in the school setting. Sommer [12] examined the menstrual experiences of older aged girls (16–19 year olds), raising concerns about the inadequate structural facilities of schools to cope with schoolgirls’ menstruation in Tanzania. Our study captured the views of predominantly 14 year old girls, many of whom had recently transitioned through menarche. We also included information from six FGDs with parents and community members to complement the data. Although school was an important part of our focus, we also wanted to understand girls’ perspectives on menstruation in their daily lives. We have identified insights into the consequences resulting from girls’ lack of preparation and knowledge on menstruation, and the impact this has on their lives. Secrecy around menstruation limits their ability to share experiences, leaving them to cope alone.

### Resources and Knowledge as Key Influences on Girls’ Experiences

This study was conducted in an impoverished area of Kenya: Nyanza is one of the poorest provinces, and Siaya is one of the poorest areas within the province [38], with a mean wealth index ranging from KSH 35,863 to KSH 42,788 (approximately U.S. $600–U.S. $700) [39]. Locally branded pads cost ∼ US$1 per pack, with up to two packs required per menses; such costs compete with essential household needs. Many girls are thus obliged, through lack of resources, rather than the unavailability of suitable menstrual products or lack of knowledge, to improvise with clearly inadequate methods of menstrual protection. While the girls questioned show great fortitude in finding ways to resolve their menstrual difficulties, some solutions adopted expose them to physical and sexual harm, as well as leading to daily struggles to manage their menstrual period adequately. Instead of menarche engendering affirmation of their ‘rite of passage’ into womanhood, it is largely viewed as a physical difficulty that when mismanaged leads to stigma, shame, and risks of sexual harm. Participants did not discuss any Luo traditions, such as girls moving to grandmothers (*pims*) homes (*siwindhe’s*) to be guided in social knowledge prior to marriage [30], however, mothers mentioned old traditions of advice from older women no longer occurred.

Our study corroborated findings that girls are ill prepared for menstruation, demonstrated in both higher [18], [40], [41] and lower income countries [23], [42]–[45]. Despite girls’ shock of experiencing their first menstruation their resilience was striking. As a minimum, girls need to be given information about menstruation before they reach menarche, and to have a basic understanding of the changes to their body that will happen on reaching puberty.

Research has shown that ignorance and lack of preparation around puberty and menstruation perpetuates myths, and leaves girls vulnerable to feelings of shame, and low self-esteem [46]. Furthermore, they appear to be vulnerable to coercion from boys and men, resulting in early sexual debut [46]. Large scale programmes such as Families Matter [47], have shown that interventions which enhance parent-child sexuality communication and use evidence-based curricula on health and sexual guidance can be implemented on a wide scale through government health and school structures with success. These programmes can improve knowledge and understanding of reproductive health. Expanding such programmes to ensure puberty education would help to address the deficit in knowledge and understanding that these young girls have about their own bodies.

Despite being a normal bodily function, menstruation was rarely discussed openly, if at all. Our separate discussions with parents corroborate this, a mother may only became aware her daughter was menstruating from clues such as cloths going missing, more frequent and private bathing, and noticing that she would distance herself from others. This illustrates how some girls proceed through menarche with minimal understanding of what is happening to them, and lacking reassurance and emotional support, or sufficient guidance on menstrual hygiene until after they have experienced a number of cycles. We suggest this may be too little, too late.

### Secrecy, Health and School Attendance

The need for secrecy has been documented in a number of studies, and was clearly a key issue for our girls. The improvised materials many of the girls have had to resort to because they, or their family, had little means to purchase sanitary pads, made secrecy even harder to achieve as most of the materials they used lack absorbency. Fear of leakage thus dominated the girls’ thoughts during their menstrual cycle. However, using traditional materials such as old cloths may add to the secrecy and shame. Girls described having to wash reusable cloths in secret, staying up late and getting up early to ensure no one sees their cloths, a finding reported elsewhere [44], [48], [49], and which adds further stress to their young lives. Moreover, researchers in Egypt report that reusable materials such as cloth, are often unhygienic due to poor washing practices, further compounded by drying secretly, in damp, dark or unhygienic places [50], a practice reported by girls in our study and similarly documented by Forde [51].

Girls showed great resourcefulness in finding materials if pads were unavailable; however, they identified a number of issues in addition to lack of absorbency associated with using improvised materials. These included irritation, chafing, restricted movement, and harm to their health. While urinary or reproductive tract infections (RTI) are reported to be causally associated with poor menstrual hygiene [52]–[54], evidence of this association in the literature remains elusive. Adolescent girls in Rajastan using ‘unsafe’ menstrual practices were estimated to have a three-fold higher odds of a symptomatic self-reported RTI compared with girls using ‘safe’ practices [44], and American women on military duties more frequently complained of vaginal irritation when unable to maintain hygiene [55]. Bacterial vaginosis was not associated with any particular menstrual items or menstrual hygiene in Gambian women complaining of vaginal itching and/or discharge [56]. Sex during menstruation has long been recognized for its potential contribution to HIV transmission [57], however its prevalence and risk in particular subgroups, including adolescent girls, has received scant attention [58]. Scientific evidence of the safety of poor menstrual hygiene is required to better understand the risks girls take in relation to their reproductive health.

Reproductive and sexual health threats such as teen pregnancy and HIV are key causes of school drop-out among schoolgirls in SSA [5], [34], [59]; however, further literature implies a direct association with menstruation [12], [23], [24], [49], [60]. The World Bank estimates poor menstrual management results in four days missed schooling every month, translating into a total loss of 10–20% of girls’ school days [11], [25]. While our baseline findings qualitatively demonstrate that menstruation appears to adversely affect both school attendance and engagement, clarity on the extent of this problem is needed as girls tended to report they themselves always attended school, but acknowledged that ‘other girls’ missed school (see limitations). Parents corroborated that there were issues with absenteeism. Girls particularly noted the impact inadequate menstrual management had on their academic engagement, and while this has been reported elsewhere [23], [49], girls in our study detail the restrictions it places on their interactions with teachers. Some girls appeared to fear teachers, and although some girls turned to them for help, not all felt that they could. This could perhaps be anticipated in a culture whereby teachers and adults are respected as figures of authority. Girls are further disadvantaged in school by the punishments they receive; keeping menstruating girls in school reduces their opportunity to change or bathe, causing physical and psychological discomfort and pain, punishing them still further.

### Menarche and Interpersonal Relationships

As stated, the FGDs were dominated by girls need for secrecy and the fear of leaks, also reported elsewhere in Kenya [23]. Should others become aware a girl was menstruating, the girl would become (or was fearful of becoming) a figure of fun, being laughed at or teased. That this was so dreaded by the girls is perhaps indicative of it being seen as a form of emotional bullying. We wonder if such bullying is the first step on the path towards gender abuse which females in this region become accustomed to. The context in which our study was set shows particularly high rates of gender-related physical, sexual and emotional violence, which appears to be an accepted part of life for women. In Nyanza Province, 36% of females report having experienced physical violence in the past 12 months, including 17% of 15–19 year olds. Within the national demographic and health survey, 56% of females believe a husband is justified in beating his wife for minor misdemeanours [36]. We surmise the teasing and humiliation displayed toward girls struggling with menstrual problems is an example of this social norm. Few girls report empathy from friends or classmates, and fear seeking help from teachers, or other figures of authority.

Once girls reach menarche, boys, and also men, appear to view them as sexually mature, placing the girls at risk of sexual advances, including coercion. While some studies have reported that physical maturation of girls triggers interest by boys and men [12], [21], [61], [62], other studies imply evidence of menarche through leakage is a precursor [12], [23]. Some girls in our study believed physical evidence of puberty (i.e. breast development) signals sexual attention, but did not connect overt leaking with sexual provocation. Forced sex is common among Kenyan girls, with 12–21% of Kenyan females reporting their first sex was non-consensual [36], [63], [64]. Significantly higher rates (∼40%) were reported among girls in western Kenya when given the opportunity to provide information confidentially through a computer survey [65]. Forced sex at debut is associated with an increased risk of STI, including HIV, and psychological stress [66], [67]. Rape and exploitation is described as a ‘*reality for youth*’ in the African setting [68]. One finding of concern in our study, raised as an issue by just a few girls, related to parental views of their daughters’ maturation. Despite their young age, a few girls acknowledged that society considered they were ready to be married or were ripe for sexual activity including, according to 2 girls and one mother, that they ‘*may be taken advantage (of)’* by their own fathers, suggesting it was not an aberrant finding, and which is also supported by other research conducted in Kenya [68].

Narratives suggest that a lack of disposable cash to purchase adequate menstrual protection is a particular hardship for these girls. Forde [51] similarly describes the struggles of young girls each month in their quest to find resources to purchase sanitary pads. As a consequence, some girls in our study appear to have participated in transactional sex in order to have money to acquire sanitary pads. In Uganda, money or transactional items are considered a ‘token of appreciation’, showing that girls were valued and respected by partners who provide for them [69]. This value placed on the girls’ sexual activities was not demonstrated in our current study. Girls who engaged in transactional sex did not appear to be viewed as immoral, however, but were accepted within the social norm. Girls were quite practical in their responses stating: ‘*because you need help, so you will just engage yourself into sex’; ‘you pay him with your vagina’* and *‘you play sex with him’.* The acceptance of the situation leads us to suggest that sex in return for gifts or money was a social norm.

### Study Limitations

We have a number of important caveats regarding interpretation of these focus group discussions. They were carried out in schools, where pupils are taught by teachers to always raise their hand and stand up before speaking. Consequently, despite our encouragement not to, discussions began formally in this manner before slowly lapsing into more natural conversations. This did, nevertheless, give the moderator an opportunity to draw out responses from the naturally quieter girls, thereby involving all in the discussions. We also consider it likely there was peer pressure from more dominant girls, causing a social desirability effect. For example, when the first girl responding reported the use of pads, subsequent girls then reported also using pads, until probed directly. However, when asked what ‘*others*’ or ‘*their friends*’ used, pads were rarely mentioned. Conducting this study within schools also limits our findings to school-attending girls. Moderator effect might also have occurred particularly during discussion on school absenteeism. It was rare for girls to admit they missed school, yet it was common for them to report ‘*other’* girls did so. The positionality of the moderators may also have impacted on the girls’ responses, as adults they were in a position of authority and treated respectfully by the girls. This may have presented a barrier to girls talking openly – however we feel this was only evident at the start of the FGDs, during the course of the discussions most of the FGDs became vibrant, with the girls opening up seemingly keen to contribute their views. The moderators were local to the area, spoke Luo, and were young women which would have helped to mitigate these constraints. By acting as observer and discussing the individual FGDs with the moderators, it was intended that issues of positionality with respect to socio-demographic and cultural issues would be reduced and allow greater understanding of the findings within the current context. That parent FGDs independently corroborated many of the findings from the girls’ FGDs serves to reassure us that our findings have consistency. Discussions could not fully explore the myriad of issues arising, due to constraints on time, and our concern girls would lose concentration or interest. Topics such as sex during menstruation, and HIV risks were thus not sufficiently explored. We resolved this partially by taking an iterative approach, repeating new focus group discussions at a later date on key findings identified.

## Conclusions

Young girls face emotional and physical challenges when experiencing menarche in this rural African population. Our findings add to the limited literature identifying girls’ unmet needs in resource-limited environments. While their acceptance and ability to overcome difficulties demonstrates resourcefulness, solutions used are suboptimal and sometimes hazardous. Girls are ill prepared for menarche, unable to share or seek reassurance, and transition through menarche mostly alone. Interventions to address these issues, as a package of measures, are required to improve the quality of girls’ lives, and reduce pressures which place them at physical and sexual harm. At community level, strategies are needed to improve communication between girls and their parents or guardians, and at school, with teachers. Structural components of schools need strengthening, to facilitate safe places for girls to clean and change. Access to better, low cost, and sustainable sanitary protection would enhance girls’ lives and reduce barriers to their engagement with schoolwork. In-depth quantitative research is required to measure risks associated with poor menstrual management, school absenteeism and engagement, exposure to sexual risks, and identify cost-effective solutions.
